# Consistent concentrations of critically endangered Balearic shearwaters in UK waters revealed by at‐sea surveys

**DOI:** 10.1002/ece3.7059

**Published:** 2021-01-26

**Authors:** Jessica Ann Phillips, Alex N. Banks, Mark Bolton, Tom Brereton, Pierre Cazenave, Natasha Gillies, Oliver Padget, Jeroen van der Kooij, James Waggitt, Tim Guilford

**Affiliations:** ^1^ Department of Zoology Oxford University Oxford UK; ^2^ Natural England Exeter UK; ^3^ RSPB Centre for Conservation Science Royal Society for the Protection of Birds Sandy, Beds UK; ^4^ MARINElife Northampton UK; ^5^ Plymouth Marine Laboratory Plymouth UK; ^6^ Centre for Environment, Fisheries & Aquatic Science (Cefas) Lowestoft UK; ^7^ School of Ocean Sciences Bangor University Menai Bridge UK

**Keywords:** Balearic shearwater, critically endangered, distribution, generalized additive model, random forest, seabird

## Abstract

**Aim:**

Europe's only globally critically endangered seabird, the Balearic shearwater (*Puffinus mauretanicus*), is thought to have expanded its postbreeding range northwards into UK waters, though its at sea distribution there is not yet well understood. This study aims to identify environmental factors associated with the species’ presence, map the probability of presence of the species across the western English Channel and southern Celtic Sea, and estimate the number of individuals in this area.

**Location:**

The western English Channel and southern Celtic Sea.

**Methods:**

This study analyses strip transect data collected between 2013 and 2017 from vessel‐based surveys in the western English Channel and southern Celtic Sea during the Balearic shearwater's postbreeding period. Using environmental data collected directly and from remote sensors both Generalized Additive Models and the Random Forest machine learning model were used to determine shearwater presence at different locations. Abundance was estimated separately using a density multiplication approach.

**Results:**

Both models indicated that oceanographic features were better predictors of shearwater presence than fish abundance. Seafloor aspect, sea surface temperature, depth, salinity, and maximum current speed were the most important predictors. The estimated number of Balearic shearwaters in the prediction area ranged from 652 birds in 2017 to 6,904 birds in 2014.

**Main conclusions:**

Areas with consistently high probabilities of shearwater presence were identified at the Celtic Sea front. Our estimates suggest that the study area in southwest Britain supports between 2% and 23% of the global population of Balearic shearwaters. Based on the timing of the surveys (mainly in October), it is probable that most of the sighted shearwaters were immatures. This study provides the most complete understanding of Balearic shearwater distribution in UK waters available to date, information that will help inform any future conservation actions concerning this endangered species.

## Graphical abstract

Areas with consistently high probabilities of shearwater presence were identified at the Celtic Sea front. Our estimates suggest that the study area in southwest Britain supports between 2% and 23% of the global population of Balearic shearwaters. This study provides the most complete understanding of Balearic shearwater distribution in UK waters available to date, information that will help inform any future UK conservation actions concerning this endangered species.

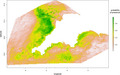

## 1 INTRODUCTION

With global population estimates ranging from 10,000 to 30,600 individuals (Arcos, 2011a; Arroyo et al., 2016; Ruíz & Martín, 2004), demographically informed population viability models suggest that without action Balearic shearwaters (*Puffinus mauretanicus*, Lowe 1921) are likely to face global extinction in the next few decades (Genovart et al., 2016; Oro et al., 2004). Their relatively small and apparently rapidly declining population (Meier, 2015), restricted breeding range, and very low survival rates compared to similar species (Genovart et al., 2016) make them the only globally critically endangered seabird in Europe (BirdLife International, 2018).

Balearic shearwaters face a number of threats including insufficient habitat for breeding, predation by introduced species, pollution (Costa et al., 2016), and decreasing prey populations (Boué et al., 2013). Notwithstanding the publication of the Action Plan to protect Balearic shearwaters by the EU in 2011 (Arcos, 2011a, 2011b), few threats to the species have been comprehensively addressed, and the global population continues to decline (Genovart et al., 2016; Oro & Guilford, 2017). Bycatch is a leading cause of loss (Cortes et al., 2017; Genovart et al., 2016; Louzao, Igual, et al., 2006; Oro et al., 2004), so reducing fisheries bycatch is a vital part of comprehensive plans to stop losses and start recovery (Abelló & Esteban, 2012; Cooper et al., 2003; Genovart et al., 2016; Louzao, Igual, et al., 2006; Louzao et al., 2011; Oro et al., 2004). Reducing bycatch could increase immature survival rates from 0.4 to 0.6, resulting in an increase in the population growth rate from 0.856 to 0.972 (Genovart et al., 2016). However, the distribution of immature shearwaters is not well understood, so conservation measures have not been specifically targeted at immatures anywhere across their range.

Balearic shearwaters breed on the Balearic Islands (Louzao et al., 2012) between February and May (Arcos, 2011a, 2011b; Guilford et al., 2012; Louzao, Hyrenbach, et al., 2006; Ruíz & Martín, 2004), and spend the postbreeding months in Atlantic waters off Portugal (Guilford et al., 2012; ICNF, 2014; Oppel et al., 2012; Ramírez et al., 2008), Spain (Mouriño et al., 2003) and France (Février et al., 2011; Yesou, 2003) with part of the population also in UK waters. Breeding birds leave the Mediterranean in late May to June to migrate into the Atlantic (Guilford et al., 2012) and return in late September to early October (Meier et al., 2015) through the Strait of Gibraltar. Occasional sightings of putative Balearic shearwaters off the English coastline have been reported since 1868 (as “Levantine shearwaters” *Puffinus puffinus mauretanicus* Wynn, 2013), but starting in the 1990s there has been an apparent increase in the number of Balearic shearwaters in northwest European coastal areas in the postbreeding period (Jones et al., 2014; Wynn & Brereton, 2009; Wynn & Yésou, 2007), particularly in northwest France (Jones et al., 2014) and southwest UK (Darlaston & Wynn, 2012; Jones et al., 2014; Wynn & Yésou, 2007), with hundreds seen off Portland (Wynn & Yésou, 2007) and occasional sightings of hundreds off Brittany (Yesou, 2003). While this apparent increase could be due to improvements in identification and increased observer awareness (Votier et al., 2008), it could also be a consequence of increasing sea surface temperatures (Luczak et al., 2011; Wynn et al., 2007) and associated changes in prey distributions (Jones et al., 2014; Luczak et al., 2011; Wynn et al., 2007, 2008), in particular, increases in anchovy and sardine populations (Alheit et al., 2012; Beare et al., 2004)—species previously limited to the Iberian and Mediterranean regions.

During the breeding season, Balearic shearwaters favor shallow shelf and near‐shore areas with thermohaline fronts close to the colony (Louzao, Hyrenbach, et al., 2006), but little is known about their preferred habitat in nonbreeding seasons (Louzao, Hyrenbach, et al., 2006). Tracking data from breeding birds have not yet revealed movements further north than Brittany (Guilford et al., 2012; Meier et al., 2017), so our knowledge of their spatial distribution in UK waters is incomplete. We also have no published estimate of Balearic shearwater abundance in UK waters.

While the movements of seabirds at sea are driven by prey (Ainley et al., 2009; Fauchald & Erikstad, 2002; Fauchald et al., 2000), a wide range of environmental factors have been found to predict seabird distributions (Cox et al., 2018), including oceanographic features, bathymetric features, primary productivity, sea surface temperature, distance to colony, and fishing activities. Habitat models that integrate these environmental characteristics to predict seabird distribution have facilitated the identification of areas critical to seabird protection (Lascelles et al., 2012; Nur et al., 2011; Oppel et al., 2012; Waggitt, Evans, et al., 2020). Generalized Additive Models (GAMs)—which allow the estimation and use of nonlinear and nonparametric relationships between species presence and predictive environmental variables (Hastie & Tibshirani, 1990; Wood, 2017)—are widely used to explain the distribution of seabirds at sea (Peron et al., 2013; Scales, Miller, Embling, et al., 2014; Virgili et al., 2017). However, predictive analyses using machine learning methods such as Random Forest (RF) can handle complex interactions across many dimensions to uncover relationships beyond the reach of traditional GAM approaches and, thus, potentially provide more accurate predictions of species distributions (Evans et al., 2011). RF is emerging as a promising method for the prediction of species distributions (Fox et al., 2017; Oppel et al., 2012; Reisinger et al., 2018) that is proving competitive with the best available traditional modeling approaches (Lawler et al., 2006; Prasad et al., 2006).

This study analyses 5 years of Balearic shearwater sightings data from annual at‐sea surveys around the southwest UK, where previous studies suggest that the species is most prevalent (Jones et al., 2014; Wynn & Yésou, 2007). We aim to (a) use explanatory RF and GAMs to determine the relationship between the distribution of Balearic shearwaters and potentially predictive variables such as prey aggregation and environmental conditions; (b) use predictive RF‐ and GAM‐based species distribution models to predict the annual and average distribution of Balearic shearwaters to identify areas of relatively higher density; and (c) estimate abundance of Balearic shearwaters in the model prediction area.

## 2 METHODS

### 2.1 Data collection

#### 2.1.1 Survey details

**Figure 1:**
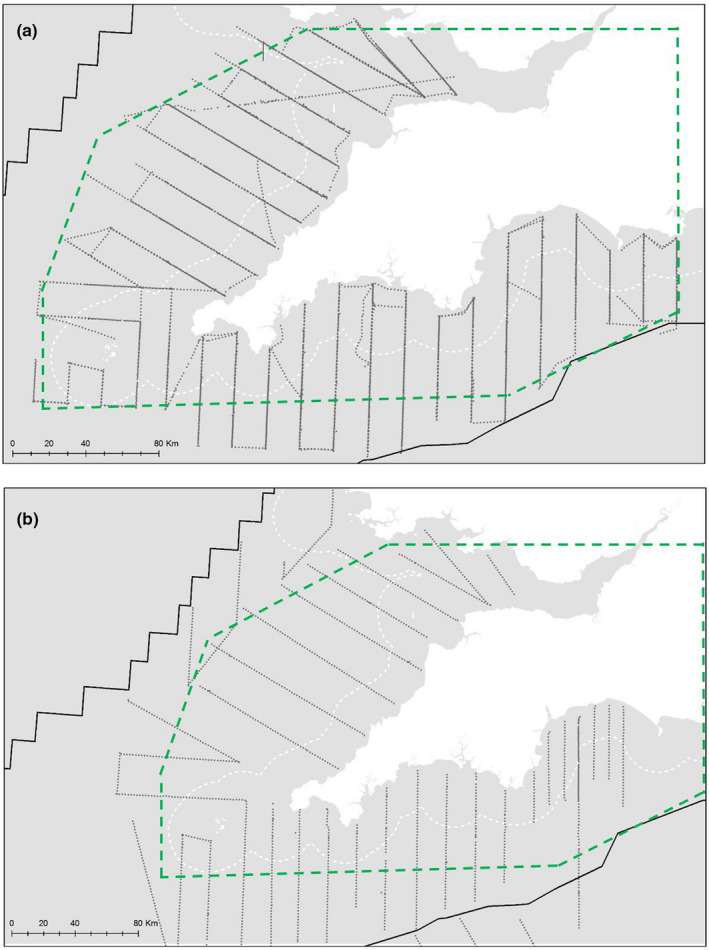
Transect lines surveyed in 2013–2016 (a) and 2017 (b) superimposed onto the area of interest where we predict Balearic shearwater presence. White dotted line indicates 12 nautical mile limit (territorial limit of England/Wales); unbroken black line indicates UK Exclusive Economic Zone limit; the green dashed line encloses the marine area in which we predict distribution and abundance

Informal sightings suggest that the waters off southwest UK (England and Wales) may be important for Balearic shearwaters (Wynn & Yésou, 2007). To investigate this, annual vessel‐based surveys of Balearic shearwater abundance were conducted between 2013 and 2017. Surveys mainly took place during October in tandem with the Pelagic Ecosystem Survey in the western English Channel and Celtic Sea, which primarily aims to map and quantify the small pelagic fish community (ICES, 2015). Most sightings of Balearic shearwaters in UK waters occur from July through October (Wynn & Yésou, 2007). The vessel followed a typical acoustic survey design along a series of parallel transects perpendicular to the coast, spaced such that spatial coverage was even (Rivoirard et al., 2000). The survey design changed in 2017 to cover slightly different transects (Figure 1a,b), but the broader area studied did not change.

#### 2.1.2 Search effort and sightings

Two methods of search were employed across the 5 year period. In all years, bespoke methods (Jones et al., 2014) were used. These methods required one observer on one side of the boat to continuously search a box 1,000 m wide, extending 300 m in front of the vessel, with 90° coverage during “effort” periods (defined as daylight hours when the vessel was not stationary or steaming between transects) grouped into 1‐min intervals. Between 2015 and 2017, additional European Seabirds At Sea (ESAS) procedures (Camphuysen et al., 2004) with 90° coverage were added to the opposite side of the transect line, giving 180° coverage of the sea. Binoculars were used to aid identification of more distant birds. Although ESAS protocol allocates bird sightings on the water into distance bands to derive detection functions for abundance estimates within 300 m of the vessel, additional observations of Balearic shearwaters were included out to 1,000 m, both to be consistent with the bespoke method and to include observations in the “band E” (i.e., >300 m from the transect) in ESAS methodology. The snapshot method (Tasker, Jones, Dixon, & Blake, 1984), used by the ESAS surveyor, was used to provide instantaneous counts of birds within 300 m ahead and 1,000 m to the side of the vessel approximately every minute (dependent on vessel speed). In years with observers on both sides of the vessel, communication between observers by radio ensured birds were not repeat counted on each side. The bird survey was suspended during trawls.

Records included the vessel's latitude and longitude, the time of observation, and the number of individuals seen. Because sightings of Balearic shearwaters were scarce, all birds on the water or in flight within 1,000 m of the transect were included in the analysis. Individuals that changed location while being observed were assigned to the first location identified. To assess whether the detection of birds decreased with increasing distance from the transect line, the distance of identified Balearic shearwaters from the observer and their angle from the observer were estimated.

Because of its influence on detectability of seabirds (Camphuysen et al., 2004), sea state was recorded continuously for inclusion as a variable in modeling, and varied between 0 and 8 on the Beaufort scale, the majority of observations (55%) recorded in sea states lower than four. Vessel speed during observations used in the analysis ranged between 0.14 knots and 16.45 knots (mean = 9.90, see Appendix 2 Figure S4). The vessel, *Cefas Endeavour*, supported observers working from a platform of 12.6 m above sea level.

#### 2.1.3 Prey variables

During bird sightings, continuous simultaneous information was collected on fish in the water‐column using fisheries acoustics. Prey data were identified using a calibrated multifrequency Simrad EK60 split‐beam echosounder (38, 120 and 200 kHz) deployed on a drop keel 8 m below the sea surface. The backscatter associated with small pelagic fish (ICES, 2015) was identified and further portioned by species using the catch composition of the nearest trawl. A pelagic (mid‐water) trawl was deployed opportunistically—when fish schools were observed on the echogram—to confirm species composition. The acoustic transect was interrupted for trawling (ICES, 2015) and resumed after completion of the trawl. A species‐specific algorithm was applied to extract the backscatter of mackerel which has a unique acoustic signature (van der Kooij et al., 2016). Prey presence and density (estimated from the Nautical Area Scattering Coefficient [NASC] value per nautical mile) were used in the analyses.

#### 2.1.4 Environmental variables

Data on salinity, sea surface temperature, and chlorophyll levels for the area of interest (latitude: 49.491 to 51.622, longitude: −6.888 to −2.003) in each of the 5 years (2013–2017) were downloaded from the Copernicus Marine Environment Monitoring Service (CMEMS). Temperature data (CMEMS, 2019b) were produced with a numeric ocean model and were available for each day on a geographic resolution of 0.25 degrees; salinity data (CMEMS, 2019a) were available in weekly averages on a 0.25‐degree grid; and chlorophyll data (CMEMS, 2019c) were available for each day on a resolution of 1km longitude by 2km latitude. We averaged the values across each survey period. Sea floor depth covering the area of interest was downloaded from the European Marine Observation and Data Network (EMODnet, 2019) with a resolution of 0.0142 degrees longitude and 0.00899 degrees latitude. Seafloor roughness, aspect and anomalies were derived from depth. Sea floor roughness identified bathymetric features associated with abrupt changes in depth, and was calculated using a terrain ruggedness index (TRI) (Wilson et al., 2007). Sea floor aspect identified bathymetry features associated with persistent depth changes in a particular direction, and was represented by the predominant slope direction. Both seafloor roughness and aspect were calculated using the “raster” (version 2.8‐4, Hijmans, 2018) package in R 3.5.3 (R core team, 2019). Sea floor anomalies identified bathymetric features associated with unusually shallow or deep depths for their location, represented by the deviance from the typical depth within that location. We modeled depth as a continuous response variable and coordinates as a continuous two‐dimensional smooth explanatory variable in a GAM with Gaussian distribution and unconstrained knots, using the “mgcv” (version 1.8‐27, Wood, 2017) and “raster” packages in R. Positive and negative residuals indicate a cell was shallower or deeper than expected for its location. Maximum current speed was the maximum depth‐averaged current speed (m/s) over a spring neap cycle extracted from an existing Finite Volume Community Ocean Model (Cazenave et al., 2016). It identifies areas of particularly strong currents, known to attract foraging seabirds in some circumstances (Waggitt et al., 2016). A stratification index (Hunter–Simpson parameter, log_10_ (*h*/*u* ^3^), where *h* is the water depth and *u* is the maximum depth‐averaged current speed) was used to identify tidal fronts (log_10_ m^−2^ s^3^ = 1.9), mixed (log_10_ m^−2^ s^3^ < 1.9), and stratified (log_10_ m^−2^ s^3^ > 1.9) water (Simpson & Sharples, 2012).

### 2.2 Analytical methods

The area of interest was divided into a grid of 1‐km^2^ cells to provide predictions of bird presence at a suitably fine spatial scale while allowing variation between cells. Because 1 km^2^ represents a small area when considering the observation methods, it was assumed that observers effectively surveyed the entire grid cell when present. Environmental data were extracted for each 1 km^2^ cell for each of the 5 years. Salinity, sea surface temperature, and chlorophyll data were estimated for each cell from its nearest neighbors, through bilinear interpolation. Sightings were attributed to cells based on the birds’ location. To reduce false absence (Oppel et al., 2012), cells were assigned “absence” only if the survey vessel spent a minimum of three minutes in the cell and no Balearic shearwaters were sighted; this requirement resulted in removing 29% of the cells with zero sightings from the analyses, but reduced the potential negative effects of false absence cells on the performance of the models (Lobo et al., 2010; Martin, 2005; Oppel et al., 2012). All processing was done in the “raster” package in R.

Three sightings were excluded because the vessel was traveling above 17 knots or spent over 15 min in the cell. Vessel speed during remaining sightings ranged from 0.14 knots to 16.45 knots (mean = 9.90). Effort was defined as the number of seconds the vessel spent in each grid cell. Sea state, the estimated wave height caused by swell and wind, was assigned to cells from observer records. Latitude, longitude, and distance to coast were calculated for the center of each cell.

#### 2.2.1 Explanatory GAM

We set the presence or absence of Balearic shearwaters as the response variable, and used a binomial distribution. We also assigned sea state as a variable to account for variations in detectability, and log transformed the fish abundances to decrease the influence of extremely high values. In order to reduce overfitting and improve the model's extrapolative abilities, GAM smoothers were constrained to four knots for each variable using REML (restricted maximum likelihood). This ensured that plausible and ecologically interpretable relationships between Balearic shearwaters and explanatory variables were produced (Lambert et al., 2017). We built GAMs for all combinations of oceanographic variables and identified the model with the lowest AIC. Maximum current speed and stratification index correlate with each other and aspect, anomalies, and roughness also correlate with each other, so only one variable from each of these two groups of variables could occur in the same GAM. Latitude and longitude were two separate variables that were only considered in models that did not include “distance to coast” because of the high correlation of these characteristics. We then built GAMs for all combinations of fish variables and compared the AIC of the fish and oceanographic models with the lowest AIC to determine which better described Balearic shearwater presence (Table 1). GAMS were performed using the “mgcv” package (version 1.8‐27, Wood, 2017) in R 3.5.3.

**Table 1:** Top five fish models and top five oceanographic models that explain Balearic shearwater presence based on the explanatory GAMs with the lowest AIC (dark grey shading indicates variables included in each model)

Variable group	Variables^a^	Oceanographic models					Fish models				
		Env.1	Env.2	Env.3	Env.4	Env.5	Fish.1	Fish.2	Fish.3	Fish.4	Fish.5
Oceanographic variables	Maximum current speed^b^										
	Stratification index^b^										
	Seafloor roughness^c^										
	Seafloor anomalies^c^										
	Seafloor aspect^c^, ^d^										
	Sea surface temperature										
	Chlorophyll										
	Salinity										
	Depth										
	Distance to coast										
Fish variables	Mackerel										
	Sprat										
	Anchovy										
	Sardine										
	Horse mackerel										
	Herring										
	Boarfish										
∆ AIC	–	0.0	2.8	5.8	7.2	11.0	161.0	161.8	162.1	162.9	163.1

^a^ All models also had sea state as a variable.

^b^ Maximum current speed and stratification index are correlated and thus cannot be used in the same model.

^c^ Seafloor roughness, seafloor anomalies, and seafloor aspect are correlated and thus cannot be used in the same model.

^d^ Orientation of the slope of the seafloor.

#### 2.2.2 Predictive GAM

Because measurements of prey were not available beyond survey areas, separate predictive models based entirely on environmental variables were constructed. To avoid making extrapolations of Balearic shearwater presence beyond the surveyed area, we restricted our prediction area to the intersection of the area of interest, the area in which we had environmental data, and the minimum convex polygon of all cells travelled to between 2013 and 2016. The transect followed in 2017 differed from previous years (Figure 1), so we did not include 2017 data in bounding the area of prediction.

As we used presence or absence as the response variable, a binomial distribution was used. We limited the analysis to variables that covered the entire prediction area (Table 2). As we modeled presence rather than abundance, we accounted for effort by excluding absent cells where the boat spent <3 min (described above). We then created GAMs from all combinations of this more limited selection of variables and identified the model with the lowest AIC. We used this model to create annual maps of the probability of the presence of Balearic shearwaters across the prediction area. We evaluated the predictive accuracy of the predictive GAMs by their area under curve (AUC), where 0.5 indicates the model has no predictive ability, 0.7–0.8 shows the model is acceptable, 0.8–0.9 indicates the model is excellent, and anything higher than 0.9 is outstanding (Hosmer & Lemeshow, 2000). We also mapped the 95% confidence intervals of the prediction. GAMs were again performed using the “mgcv” package in R 3.5.3.

**Table 2:** Variables evaluated for each of the four models (dark grey shading indicates variables included in each model)

Group	Variables	Explanatory GAM^a^	Predictive GAM	Explanatory RF	Predictive RF
Static oceanographic features	Maximum current speed				
	Stratification index				
	Depth				
	Seafloor roughness				
	Seafloor anomalies				
	Seafloor aspect^b^				
Dynamic oceanographic features	Sea surface temperature				
	Chlorophyll				
	SALINITY				
Fish abundance	Mackerel				
	Sprat				
	Anchovy				
	Sardine				
	Horse mackerel				
	Herring				
	Boarfish				
Location	Longitude				
	Latitude				
	Distance to coast				
Survey specifics	Sea state				

^a^ Fish abundance variables and oceanographic variables were not included in the same models; they were considered in separate sets of models as shown in Table 3.

^b^ Orientation of the slope of the seafloor

#### 2.2.3 Explanatory RF

Random Forest bootstrap samples the dataset, fitting a regression tree to each random subset of the data (Breiman, 2001). At each split in the tree, the data are divided in two by the value of a predictor variable, chosen from a random subset of all predictor variables. Each tree then predicts the out‐of‐bag (OOB) observations (i.e., data not used in the construction of that tree), with the errors in these predictions called OOB error. Breiman (1996) showed that OOB error is as good as the error estimate calculated from setting aside a test‐dataset that is equal in size to the training dataset (Breiman, 2001). All OOB predictions are averaged to generate predictions for each observation (Cutler et al., 2007). As RF models are nonparametric and do not assume independence, they are not affected by spatial auto‐correlation (Evans et al., 2011). RF ranks variable importance by the drop in the accuracy of the predictions when that variable is randomized (Prasad et al., 2006), a method that can identify the most ecologically meaningful variables more effectively than other methods (Cutler et al., 2007).

**Figure 2:**
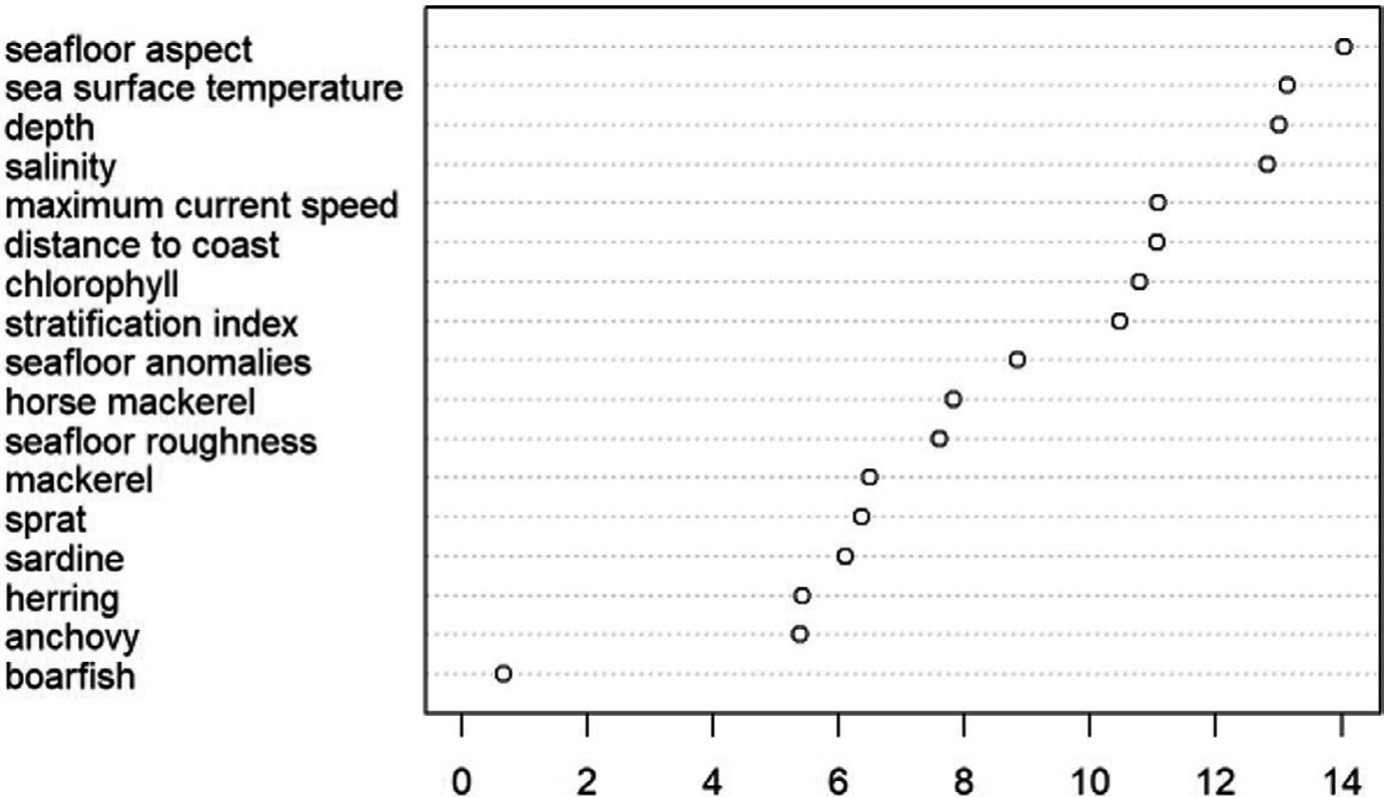
Importance of variables in the explanatory random forest, calculated from the drop in prediction accuracy when each variable is randomized. The values on the *x*‐axis have no intrinsic meaning, they represent the relative importance of the 17 variables included in the model

With the nine oceanographic variables, distance to coast, and the abundance of seven fish species as the predictor variables (Table 2), we ran a RF on our dataset using the “RandomForest” package (version 4.6‐14, Liaw & Wiener, 2002). Unlike in the GAM analyses, in this RF analysis fish abundances were not transformed as RF does not require data to be normally distributed (Evans et al., 2011), and correlated variables were not removed as RF spreads the importance of the collinear variables (Cutler et al., 2007). We grew 500 regression trees, each on a random subset of 66% of the cells. Each node in each tree split the data with a variable from a random sample of four of the 17 predictor variables. We then ranked the importance of the variables (Figure 2).

#### 2.2.4 Predictive RF

We first excluded fish abundance variables when creating the predictive model, as no data were available outside the boat transects. In order to have an unseen dataset to test the predictive accuracy of our RF model, we set aside the 2013 data, and used the 2014–2017 data as the training dataset. As there are only 179 “present” cells among the 8,107 cells with known presence or absence across the 5 years, we randomly sampled absences so that there would be the same number as presences, to reduce the class imbalance in the training dataset (Sun et al., 2009). In RF models, by default the number of randomly selected predictive variables to split each node in a tree is the square root of the number of predictive variables rounded down to the nearest integer. Using the “SuperLearner” package (version 2.0‐25, Polley et al., 2019), we built RF models with three multiples of this default (0.5, 1, and 2), compared their performance and produced an optimal weighted average, an “ensemble.”

This process was repeated with each of the remaining years held out in turn. We then used each of the five ensembles to predict Balearic shearwater presence and absence on the held‐out year, and calculated the area under curve (AUC), a metric of predictive accuracy which reflects its ability to accurately predict the unseen year. We then used all 5 years of data to create a model ensemble and used that to map the probability of presence of Balearic shearwaters in each year. The area of prediction considered is the same as that described in Section 2.2.2.

#### 2.2.5 Abundance estimate

Oppel et al. (2012) attempted to predict Balearic shearwater abundance using five methods, including RF and GAMs; they concluded that all five models had limited predictive power. Given this prior finding and the relatively low number of sightings in our dataset, we were unable to reliably map the spatial variation of Balearic shearwater abundance across our survey area. However, we were able to estimate the overall (mean) abundance in the entire survey area.

For this analysis, we only included birds sighted within 300 m of the vessel transect, as there appears to be little decline in the detectability to this distance (see Appendix 2 Figure S5) as most birds were detected in flight. We assume all birds present within 300 m of the vessel transect were detected, allowing us to use a strip width of 300 m in 2013 and 2014, and 600 m in 2015–2017 to estimate density, without correction for detectability. We estimated density by dividing the numbers of Balearic shearwaters seen within 300 m of the boat transect by the total area searched per year (i.e., the distance of the transect multiplied by 600 m or 300 m in 2013 and 2014) (Table 3). We assumed that surveys covered a broad range of habitats, and would have included habitats supporting both low and high densities of birds. Therefore, the densities calculated above should be representative of densities across the region. Thus, we estimate the number of individuals in our prediction area in each year by multiplying this density by the size of the prediction area (41,771 km^2^), the same polygon on which we predicted distribution. We derived variance of bird density by calculating the variance in bird density across days, weighted by transect distance covered each day. Multiplying the variance in density for each year by the size of the prediction area produced the variance in abundance.

**Table 3:** Estimated abundance of Balearic shearwaters in the prediction area in each year during the survey period

Year	Transect distance (km)	Birds within 300 m of transect^a^	Density (variance) (birds/km^2^)	Abundance (variance)
2013	2,232	43 [43]	0.064 (0.012)	2,682 (521)
2014	2,621	130 [80]	0.17 (0.12)	6,904 (4,925)
2015	2,414	73 [64]	0.050 (0.012)	2,106 (506)
2016	1,896	36 [33]	0.032 (0.0055)	1,322 (230)
2017	3,203	30 [26]	0.016 (0.0019)	652 (79)

^a^ Numbers in square brackets are the number of birds which were flying.

## 3 RESULTS

In total, the vessel covered transects totaling 12,366 km in 100 days of surveying across the 5 years (Tables 3 and 4). The final analysis included data on 393 birds sighted in 179 1‐km^2^ grid cells (Table 4).

**Table 4:** Details of vessel survey in UK waters and Balearic shearwaters sighted per year

Year	Survey method	Dates	Duration (days)	Number of birds	Number of 1 km^2^ cells with birds (% of cells with birds)
2013	BM	15–30 October	16	66	42 (3.2%)
2014	BM	4–17 October	14	168	61 (3.2%)
2015	ESAS	4–21 October	18	62	33 (2.0%)
2016	ESAS	4–19 October	16	84	34 (2.3%)
2017	ESAS	29 September−3 November	36	13	9 (0.5%)
Total	.	.	100	393	179 (2.2%)

Abbreviations: BM, bespoke methods; ESAS, European Seabirds at Sea.

**Figure 3:**
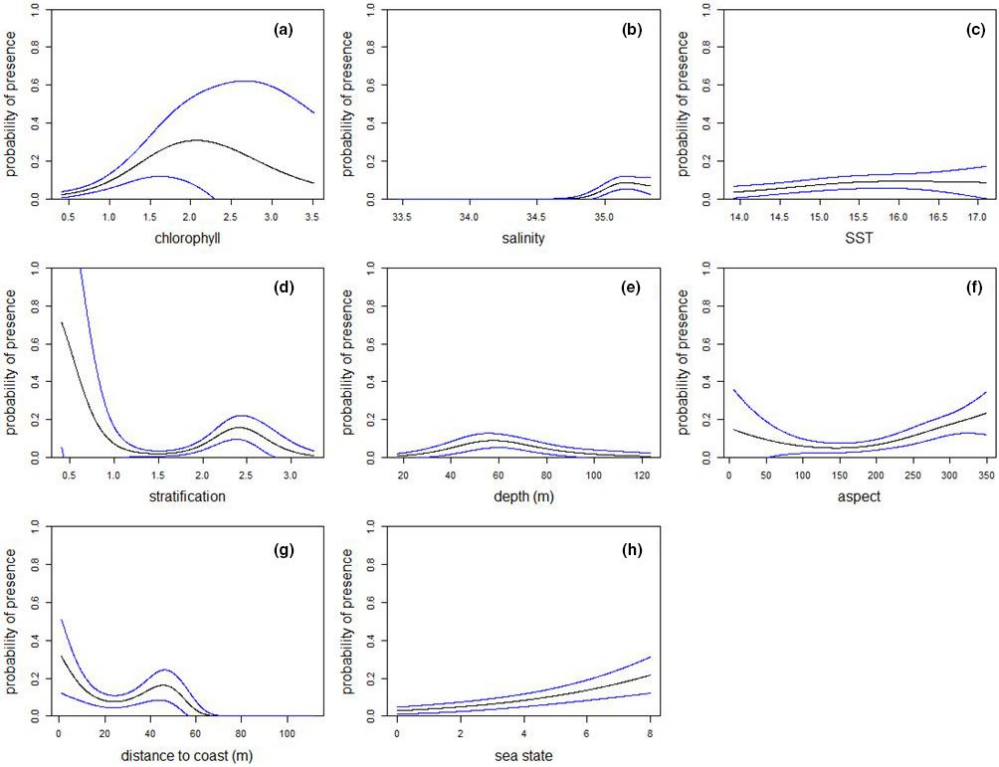
Relationship between variables retained in explanatory GAM with lowest AIC and probability of Balearic shearwater presence when all other variables are set at their mean. For (a) chlorophyll; (b) salinity; (c) sea surface temperature; (d) stratification index; (e) depth; (f) aspect; (g) distance to coast; and (h) sea state

The explanatory oceanographic GAM with the lowest AIC contained chlorophyll, salinity, sea surface temperature, depth, sea floor aspect, distance to coast, and stratification index (Figure 3, Table 1). The explanatory fish GAM with the lowest AIC contained mackerel, sprat, anchovy, horse mackerel, herring, and boarfish. The oceanographic model had a substantially lower AIC (∆AIC = 161.0) (Table 1).

The ranking of the variable importance in the explanatory RF (Figure 2) supports the conclusion of the explanatory GAMs that oceanographic variables are better at predicting Balearic shearwater presence than surveyed fish abundance. In the RF variable ranking, all oceanographic variables ranked higher than any fish species abundance, except for seafloor roughness which ranked below horse mackerel. Sea floor aspect emerged as the most significant variable followed by depth and salinity. The explanatory RF has an OOB error rate of 0.06, indicating the model has high accuracy.

**Figure 4:**
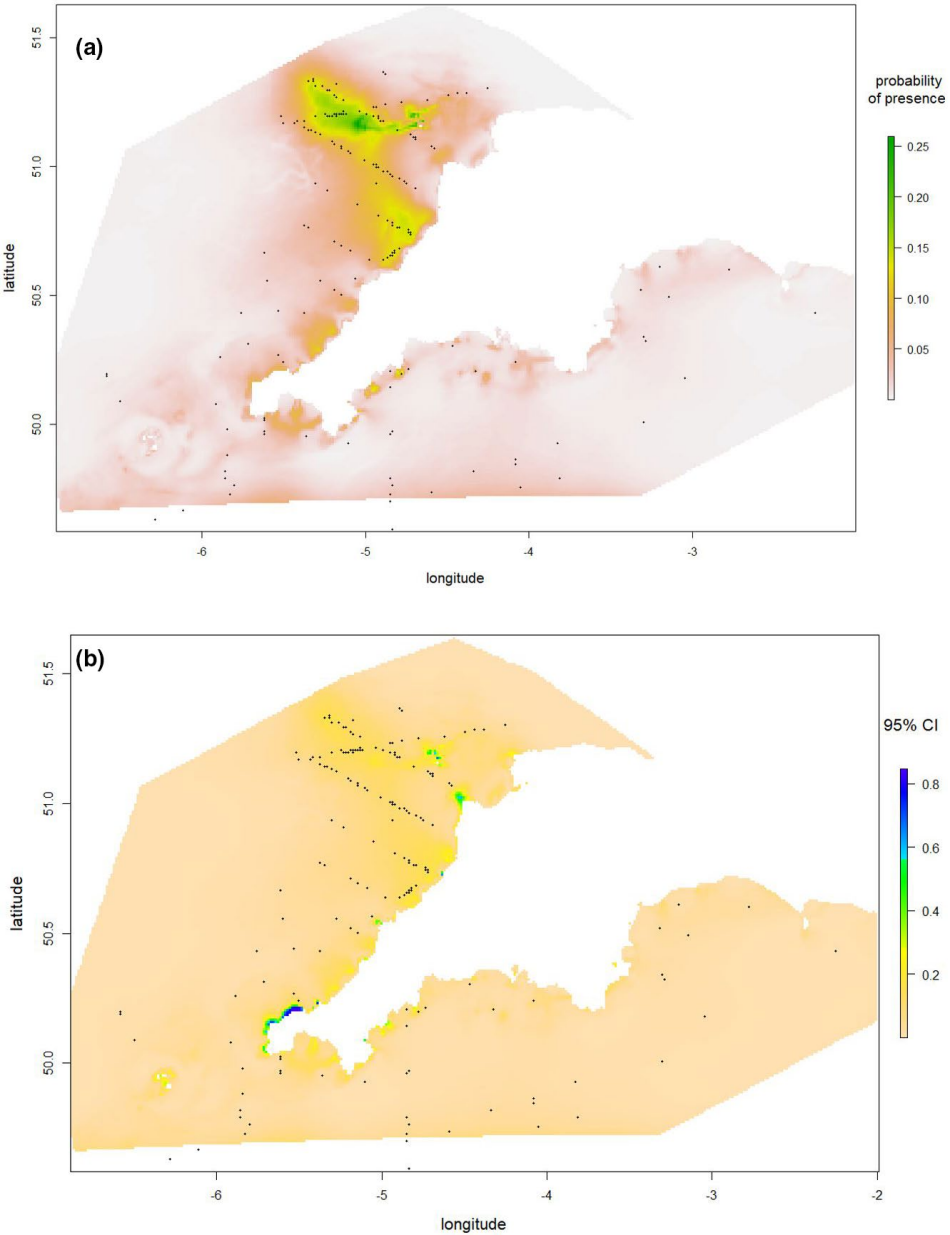
(a) Probability of Balearic shearwater presence predicted by the Generalized Additive Model averaged across 5 years, and (b) the 95% confidence intervals of the probability of presence (i.e., the difference between the upper and lower bound), with sightings superimposed on the maps (black dots)

**Figure 5:**
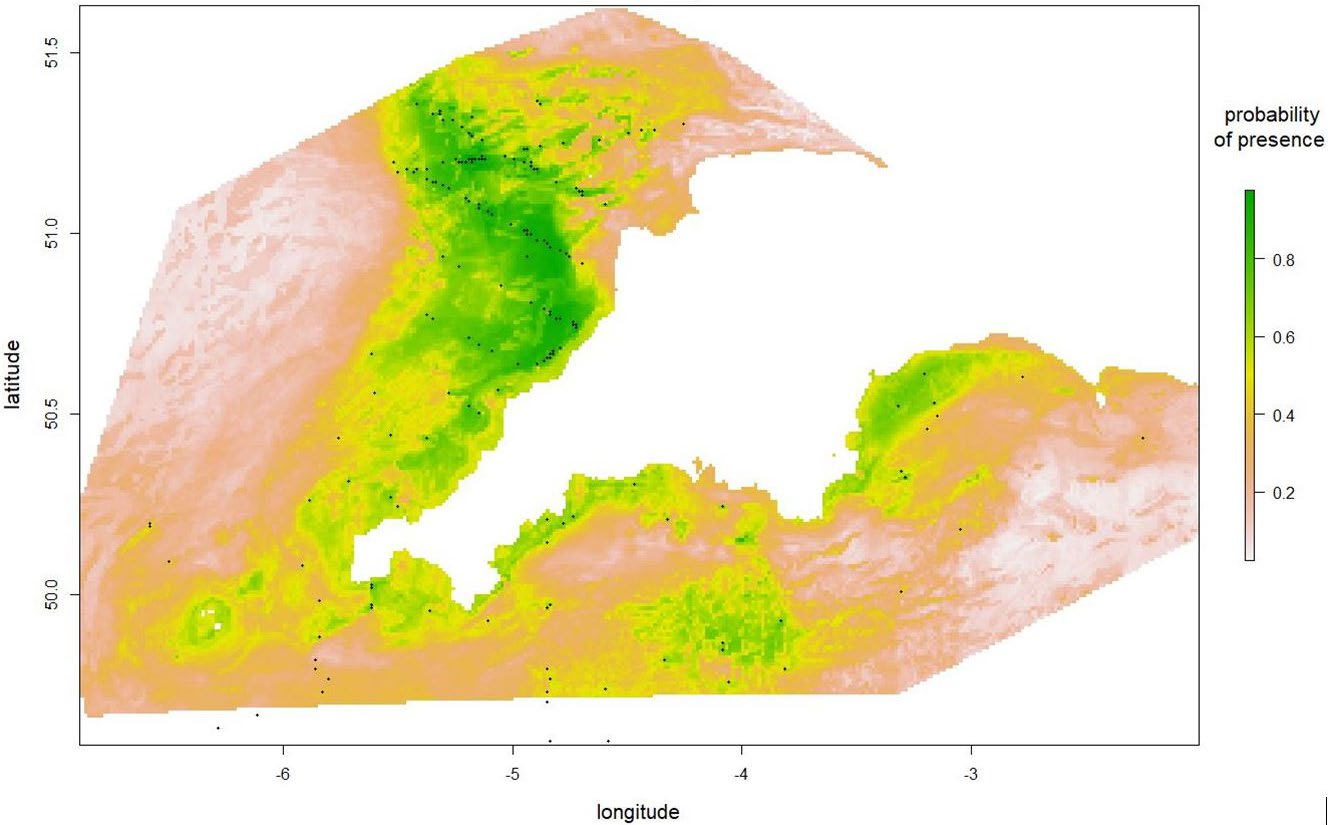
Probability of Balearic shearwater presence predicted by Random Forest model ensembles averaged across 5 years with sightings superimposed on the map as black dots

The predictive GAM retained depth, sea surface temperature, salinity, chlorophyll, latitude, longitude, sea floor anomalies, and stratification index. As shown in Figure S3, there is no evidence of residual spatial auto‐correlation. The predictive GAM has an AUC of 0.869 for 2013, 0.839 for 2014, 0.848 for 2015, 0.837 for 2016, 0.669 for 2017; the AUC was 0.849 in a model that combined all 5 years of data. The AUC for the model ensembles with each of 2013–2017 data held out were as follows: 0.78, 0.68, 0.80, 0.75, and 0.48. We used a model ensemble built on all 5 years of data to make predictive maps of Balearic shearwater distributions for each of the 5 years. The predictive maps of the GAM and RF identified similar areas of highest shearwater density (Figures 4 and 5, and see Figures S1 and S2).

The estimated number of Balearic shearwaters in the prediction area ranged from 652 birds in 2017 to 6,904 birds in 2014 (Table 3).

## 4 DISCUSSION

Our findings suggest that there is a high probability of occurrence of nonbreeding Balearic shearwaters at the Celtic Sea front (Simpson, 1976) with relatively stable interyear structure from 2013 to 2017. Both our explanatory GAM and RF models indicate oceanographic variables are better predictors of Balearic shearwater presence than surveyed fish abundance. This finding is in line with previous work on shearwaters which highlights the importance of frontal features (Scales, Miller, Hawkes, et al., 2014), including those on breeding Balearic shearwaters in the western Mediterranean (Arcos & Oro, 2002; Louzao et al., 2011, 2012). The discussion initially focuses on these findings, before mentioning the conservation implications of these results.

The distribution of seabirds should broadly overlap with the distribution of their prey (Fauchald et al., 2000). However, prey abundance did not emerge as the most important predictor of Balearic shearwater presence in our analysis (Figure 2). There are three potential explanations for this difference. First, the fisheries acoustic equipment used to assess prey abundance was mounted on a drop keel below the hull, not capturing fish abundance in the top 12 m of water and excluding the main foraging depth of Balearic shearwaters (Meier et al., 2015). Thus the prey measured could be abundant at depths less preferred or inaccessible to seabirds (Boyd et al., 2017, Waggitt et al., 2018). Second, fisheries discards—which occur irrespective of prey abundance—are a substantial part of the Balearic shearwater's diet (Arcos & Oro, 2002). Although Balearic shearwaters are known to follow trawlers, they are less likely to do so after the breeding season (Arcos & Oro, 2002) and trawler presence does not predict shearwater distribution on large spatial scales (Louzao, Hyrenbach, et al., 2006). While we did not have data on fishing activities, it would be useful for future predictive analyses to account for its impact. Third, predator and prey distributions may be mismatched at the fine scales (<1 km) used in our analyses as seabirds do not have complete knowledge of prey distribution (Gremillet et al., 2008), and prey can engage in antipredator responses (Crook & Davoren, 2014). This study reaffirms the difficulty of capturing spatiotemporal relationships between mobile marine top predators and prey (Fauchald, 2009). However, in doing so, this study supports suggestions that oceanographic variables indicative of enhanced prey availability are better predictors of marine top predator distributions than direct measurements of prey abundance (Torres et al., 2008).

Knowledge on Balearic shearwater phenology and migration suggests that most shearwaters sighted were nonbreeders. In 2013 and 2014, the mean date breeding Balearic shearwaters, tracked from Mallorca, returned through the Strait of Gibraltar was September 26th and September 22nd, respectively (Meier et al., 2015). Therefore, breeding birds would have predominantly been back in the Mediterranean before the annual surveys described here were conducted in October, which suggests that birds sighted during the surveys were not breeding birds. Sighting data from SeaWatch SW supports this hypothesis, showing that Balearic shearwaters are found in the southwest UK throughout the breeding period (Jones et al., 2014), with the highest number of sightings occurring when breeding birds were at their colonies in the Mediterranean (Guilford et al., 2012). Additionally, neither breeding birds tracked on Mallorca (Meier et al., 2015) nor Ibiza (Perez‐Roda et al., 2017) travelled as far north as UK waters, making it unlikely that birds sighted during the survey were breeding birds from other colonies displaying migratory segregation (Guilford et al., 2012; Louzao et al., 2011). Sighted birds are also unlikely to be failed breeders as failed breeders tracked on Mallorca were predominantly back before our surveys started (Meier et al., 2015). To date, the only Balearic shearwater of known provenance that has been seen as far north as our surveys were conducted was an immature (Wynn, 2013). Thus, most of the birds sighted in our surveys were probably immatures, and possibly some adults taking sabbaticals.

We identified the Celtic Sea front as an important habitat for Balearic shearwaters across years (Figures S1 and S2), mirroring findings from previous studies on Manx shearwaters *Puffinus puffinus* in the region (Waggitt et al., 2018). Tidal fronts are linked to high primary and secondary productivity, attracting large numbers of marine predators (Scales, Miller, Hawkes, et al., 2014). The interface between mixed and stratified waters at tidal fronts could also provide suitable combinations of prey density, depth, and prevalence, maximizing prey availability and providing good foraging opportunities for diving seabirds (Waggitt et al., 2018). Moreover, tidal fronts are persistent in time and space, allowing seabirds to efficiently locate these foraging opportunities from memory (Scales, Miller, Hawkes, et al., 2014). Finally, we found that Balearic shearwater presence increased at higher sea states. Flying shearwaters are probably more visible in higher winds because they are more likely to be shear soaring, intermittently presenting the observer with a large surface area as well as an alternating light‐dark coloration. Therefore, the association between Balearic shearwaters and high sea state may indicate enhanced detectability of flying birds (Waggitt et al., 2020), rather than larger abundances of birds during stormier conditions.

We found considerable interannual variation in abundance of Balearic shearwaters in southwest UK over the 5 years of the survey (2013–2017). As all static oceanographic features remained constant across years, changes in model predictions must be related to changes in dynamic oceanographic features such as sea surface temperature, salinity, and chlorophyll. Investigating interannual differences in the abundance of Balearic shearwaters requires additional studies considering oceanographic processes and associated movement of prey. However, when assessing this interannual variation from a conservation perspective, our estimates suggest that the study area in southwest UK annually supports between 2% and 23% of the global population of Balearic shearwaters (based on a global population estimate of 30,600, Arcos, 2011a). Although the survey was explicitly designed to be systematic, there were small deviations from the planned route, which could introduce some bias to our calculations. The abundance value presented is inevitably an estimate, and is susceptible to factors we could quantify variance in, as well as factors that were much harder to quantify variance in. Despite the variation, even the minimum abundance estimate (2% of world population of Balearic shearwaters) is still of global importance.

Further studies are needed to determine the threats facing the species in this area. Current knowledge of bycatch, for example, across this area is very limited, but this study will help identify the areas of greatest interest.

## 5 CONCLUSION

We have presented the most comprehensive description of Balearic shearwater distribution in UK waters currently available. We found higher probability of occurrence around the Celtic Sea front with relatively stable interyear structure. If, as evidence here suggests, this location is a key foraging ground for immature Balearic shearwaters, the area could be the primary focus should any conservation measures separately be identified. The widespread predicted distribution of Balearic shearwaters at lower probability (especially with the RF model) means pressures, perhaps including known issues in other parts of the species' range such as fisheries bycatch, may also require mitigation if discovered to be acting elsewhere in UK waters.

## CONFLICT OF INTEREST

None declared.

## AUTHOR CONTRIBUTIONS

**Jessica A. Phillips:** Formal analysis (lead); Writing‐original draft (lead); Writing‐review & editing (equal). **Alex Banks:** Conceptualization (lead); Formal analysis (supporting); Project administration (lead); Supervision (supporting); Writing‐original draft (supporting); Writing‐review & editing (equal). **Mark Bolton:** Data curation (equal); Formal analysis (supporting); Writing‐review & editing (equal). **Tom Brereton:** Data curation (equal); Formal analysis (supporting); Writing‐review & editing (equal). **Pierre Cazenave:** Data curation (equal); Writing‐review & editing (equal). **Natasha Gillies:** Formal analysis (supporting); Methodology (equal); Writing‐review & editing (equal). **Ollie Padget:** Formal analysis (supporting); Project administration (supporting); Writing‐review & editing (equal). **Jeroen van der Kooij:** Data curation (equal); Formal analysis (supporting); Methodology (equal); Writing‐original draft (supporting); Writing‐review & editing (equal). **James Waggitt:** Data curation (equal); Formal analysis (lead); Validation (lead); Writing‐review & editing (equal). **Tim Guilford:** Formal analysis (supporting); Supervision (lead); Writing‐original draft (supporting); Writing‐review & editing (equal).

## Supporting information

Appendix

## Data Availability

Data on salinity, sea surface temperature, and chlorophyll levels were downloaded from the Copernicus Marine Environment Monitoring Service, at: http://marine.copernicus.eu/services‐portfolio/access‐to‐products/. Data on sea floor depth, sea floor aspect, sea floor anomalies, and sea floor roughness were downloaded from the European Marine Observation and Data Network, at: https://www.emodnet‐bathymetry.eu/data‐products. Data on small pelagic fish distribution and abundance are available for some species at https://acoustic.ices.dk/submissions. Other data are available on request from Jeroen Van Der Kooij. 2015–2017 ESAS bird sightings data under contract to Natural England, all other bird sightings data collected by MARINELife, available on request. The raster layers of the maximum current speed and stratification index used to create the models, as well as the R code, are available on Dryad at https://doi.org/10.5061/dryad.9p8cz8wdz.
