# Supplementary material for: Consistent concentrations of critically endangered Balearic shearwaters in UK waters revealed by at‐sea surveys
Source: Ecol Evol. 2021 Jan 26;11(4):1544–57. doi: 10.1002/ece3.7059 (PMC7882943; doi:10.1002/ece3.7059)

Appendix 1

<u>Figure S.1</u> Probability of Balearic shearwater presence predicted by the Generalized Additive Model in a) 2013; c) 2014; e) 2015; g) 2016 & i) 2017, and 95% confidence intervals of these predictions for in b) 2013; d) 2014; f) 2015; h) 2016 & j) 2017.

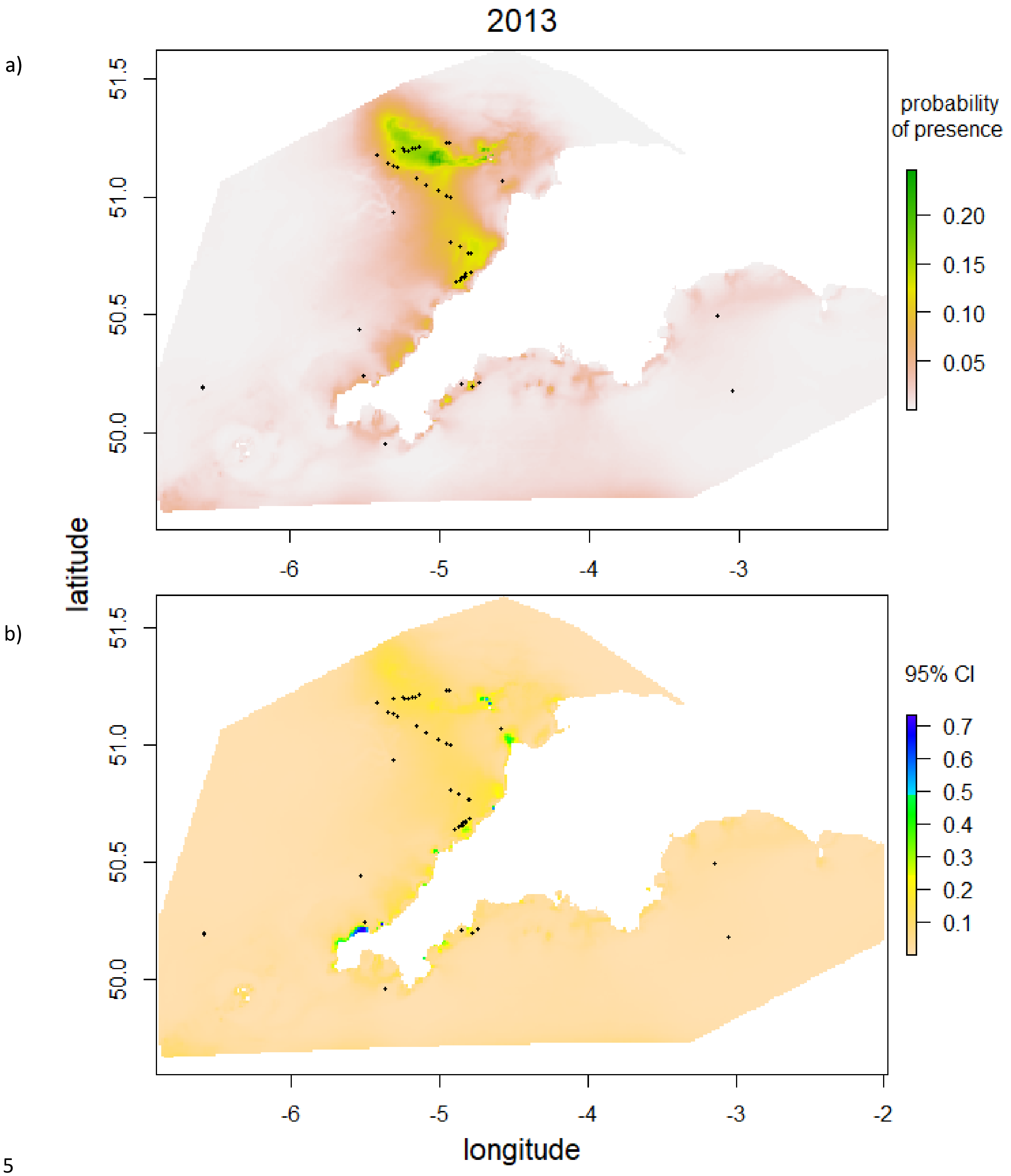

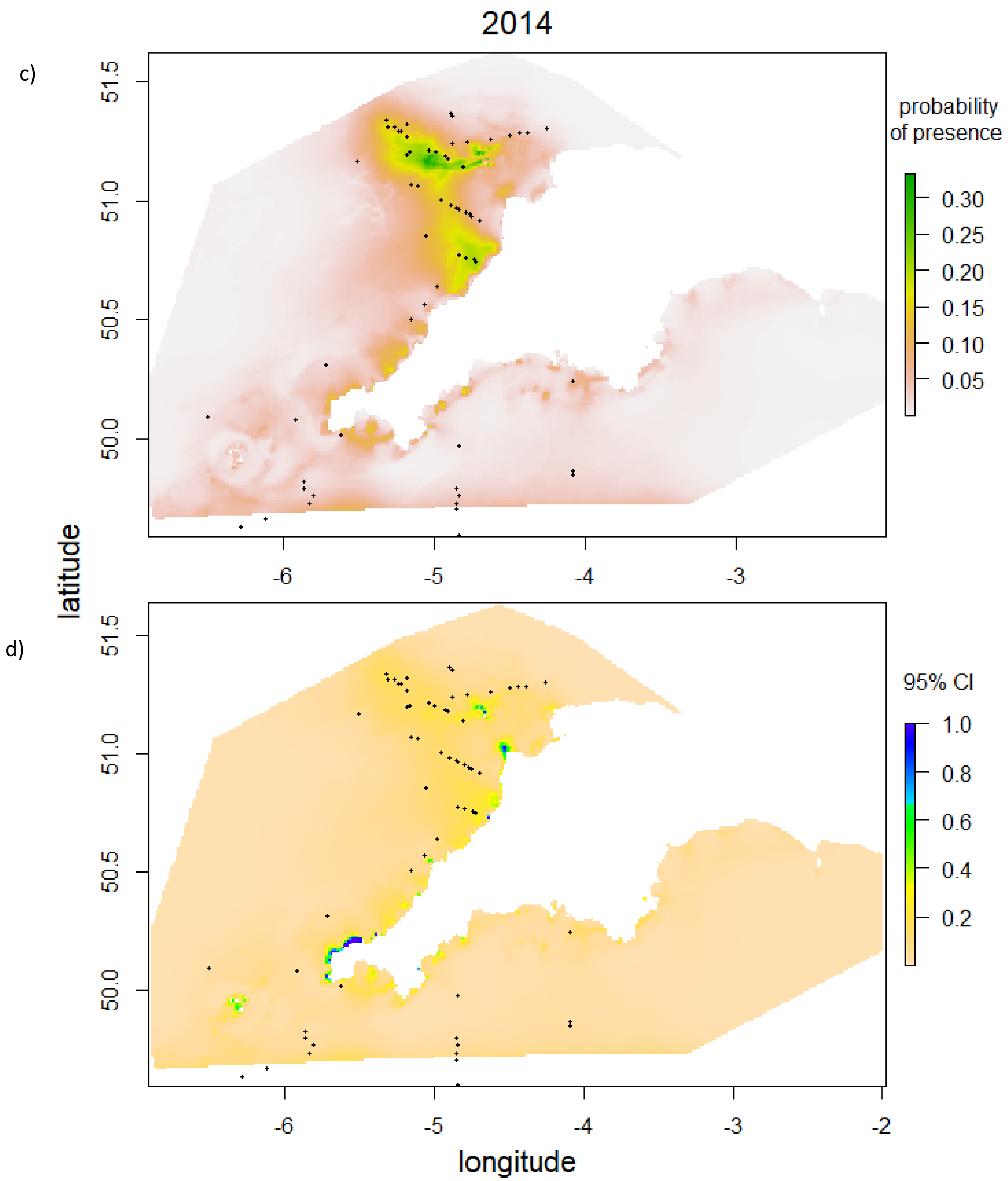
2014
c)
d)
probability
of presence
0.30
0.25
0.20
0.15
0.10
0.05
95% CI
1.0
0.8
0.6
0.4
0.2
latitude
longitude
51.5
51.0
50.5
50.0
-6
-5
-4
-3
-2

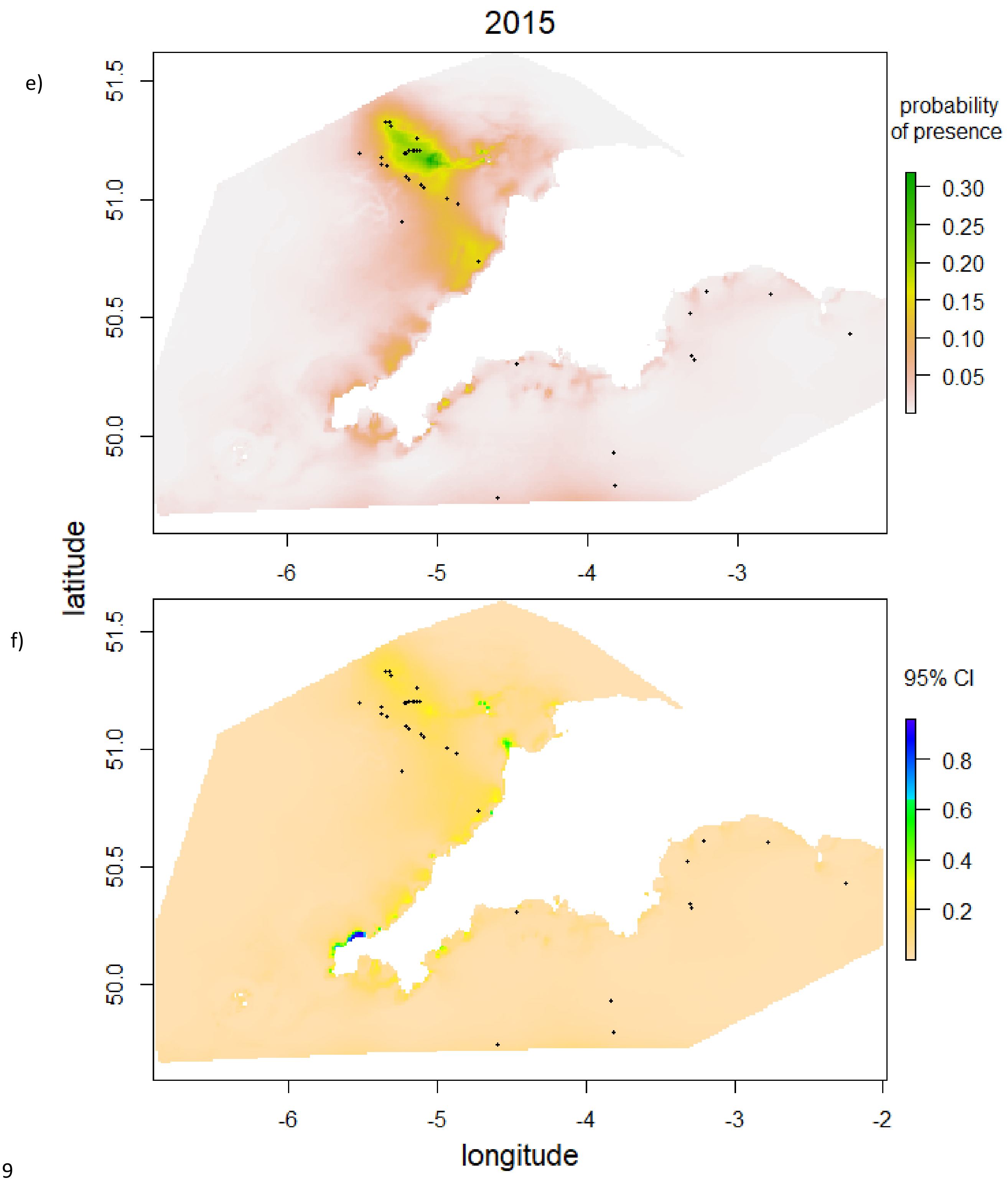
2015
e)
f)
probability
of presence
0.30
0.25
0.20
0.15
0.10
0.05
95% CI
0.8
0.6
0.4
0.2
51.5
51.0
50.5
50.0
-6
-5
-4
-3
-2
latitude
longitude

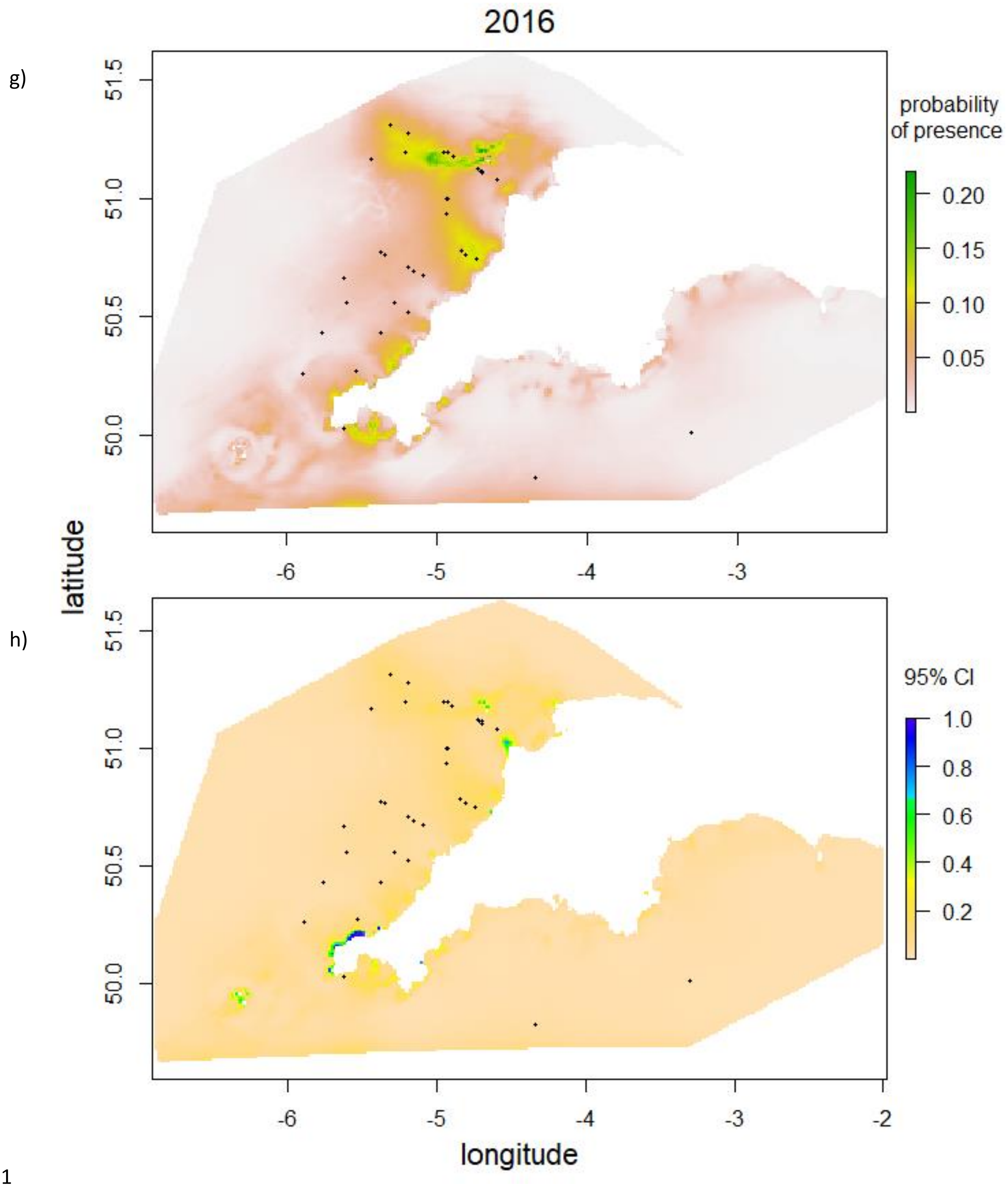
2016
g)
h)
probability
of presence
0.20
0.15
0.10
0.05
95% CI
1.0
0.8
0.6
0.4
0.2
latitude
51.5
51.0
50.5
50.0
-6
-5
-4
-3
-2
longitude

i)

j)

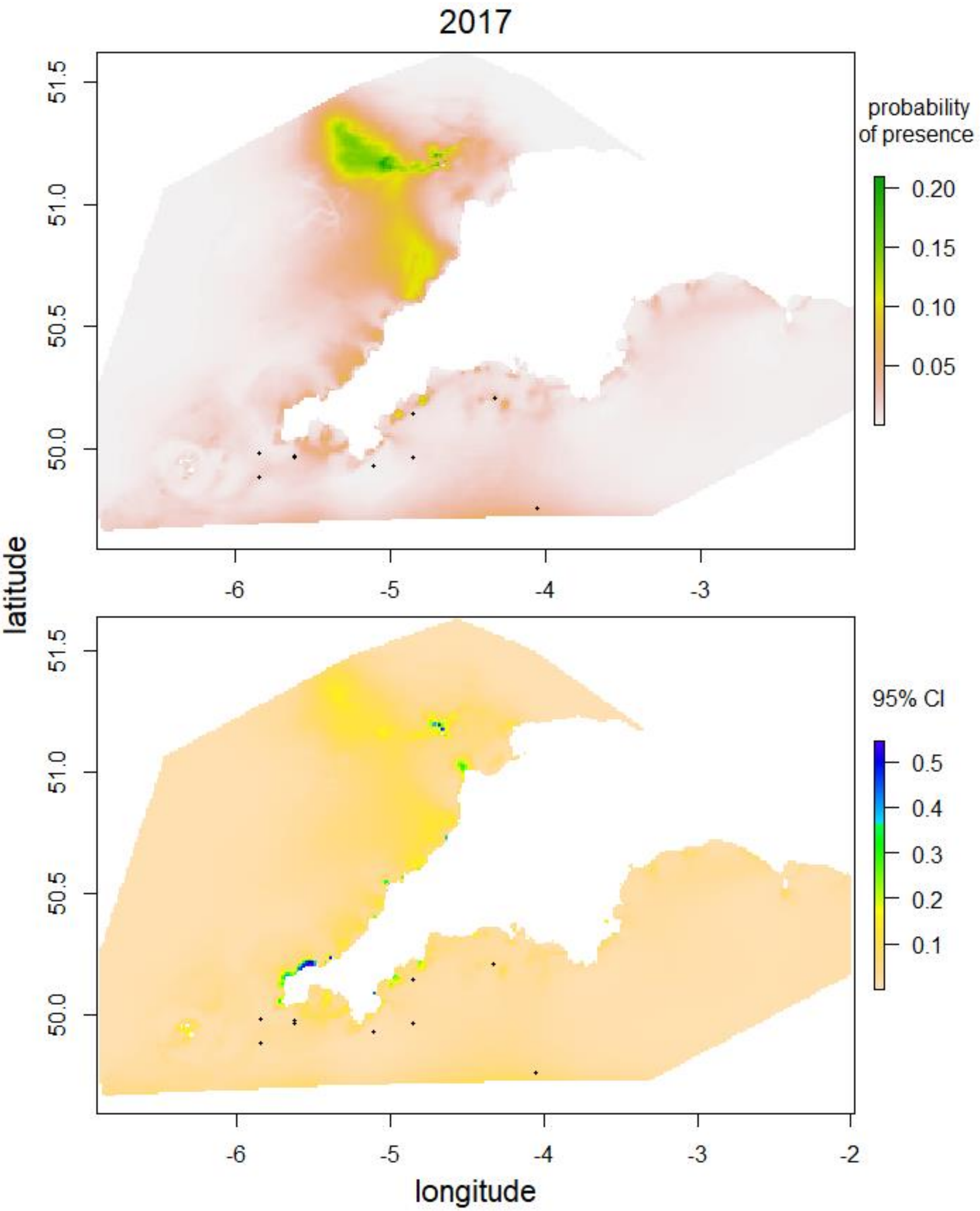

2017
probability
of presence
0.20
0.15
0.10
0.05
95% CI
0.5
0.4
0.3
0.2
0.1
latitude
51.5
51.0
50.5
50.0
-6
-5
-4
-3
-2
longitude

<u>Figure S.2</u> Probability of Balearic shearwater presence predicted by a Random Forest model ensemble for a) 2013; b) 2014; c) 2015; d) 2016 & e) 2017

(a)

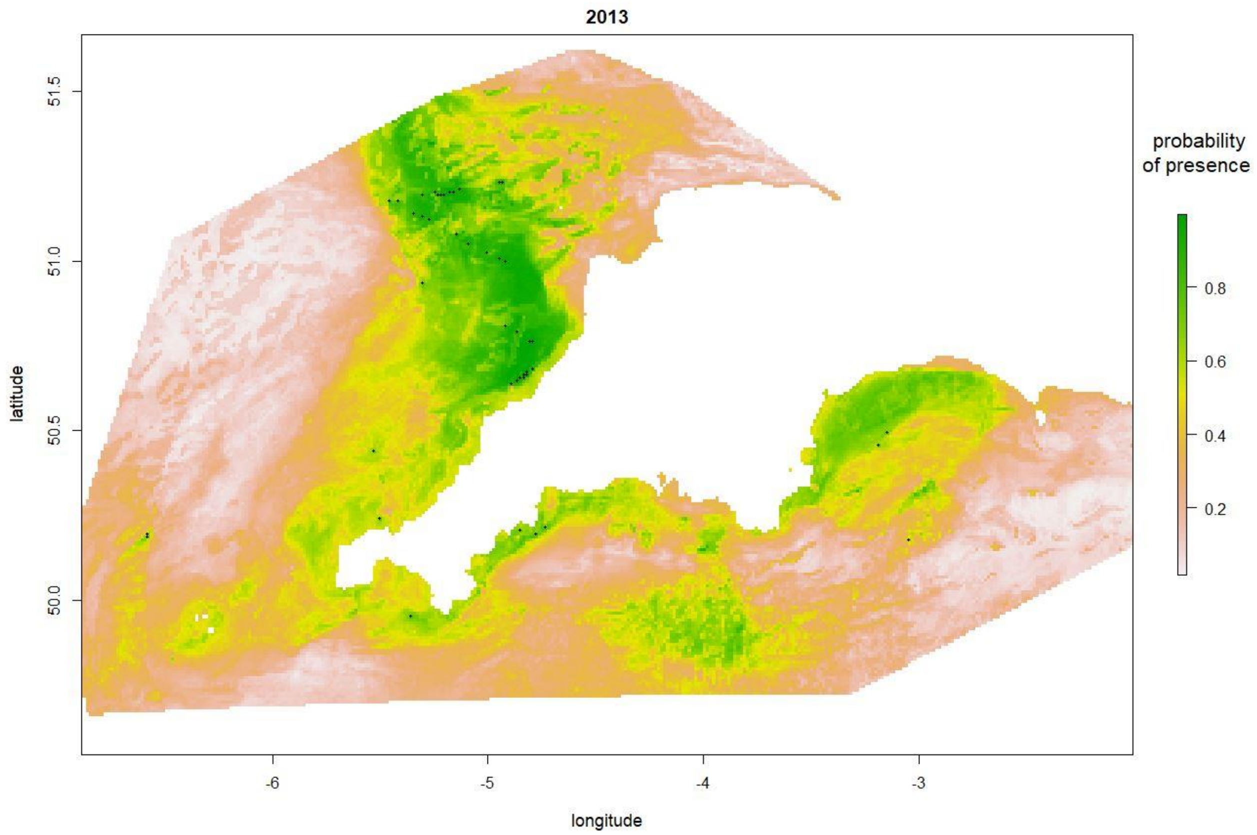

(b)

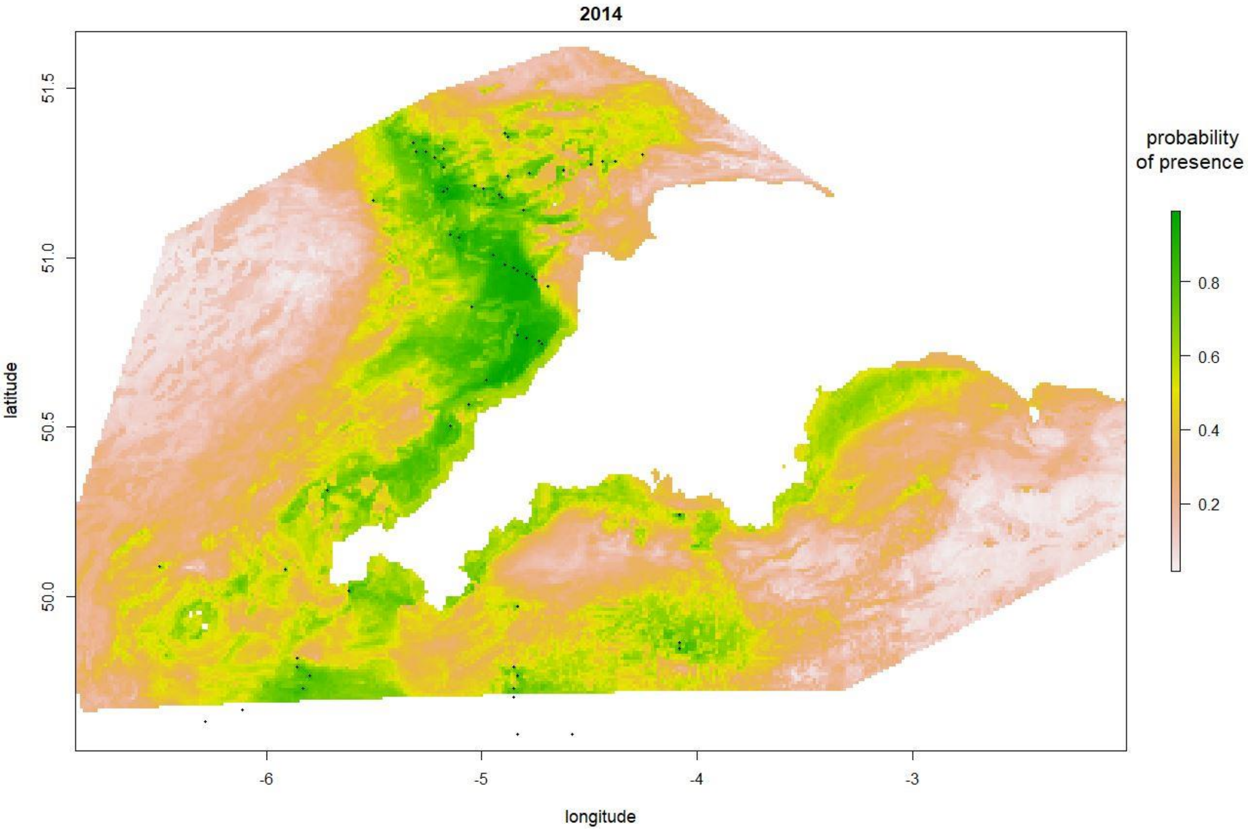
2014
latitude
longitude
probability
of presence
51.5
51.0
50.5
50.0
-6
-5
-4
-3
0.8
0.6
0.4
0.2

(c)

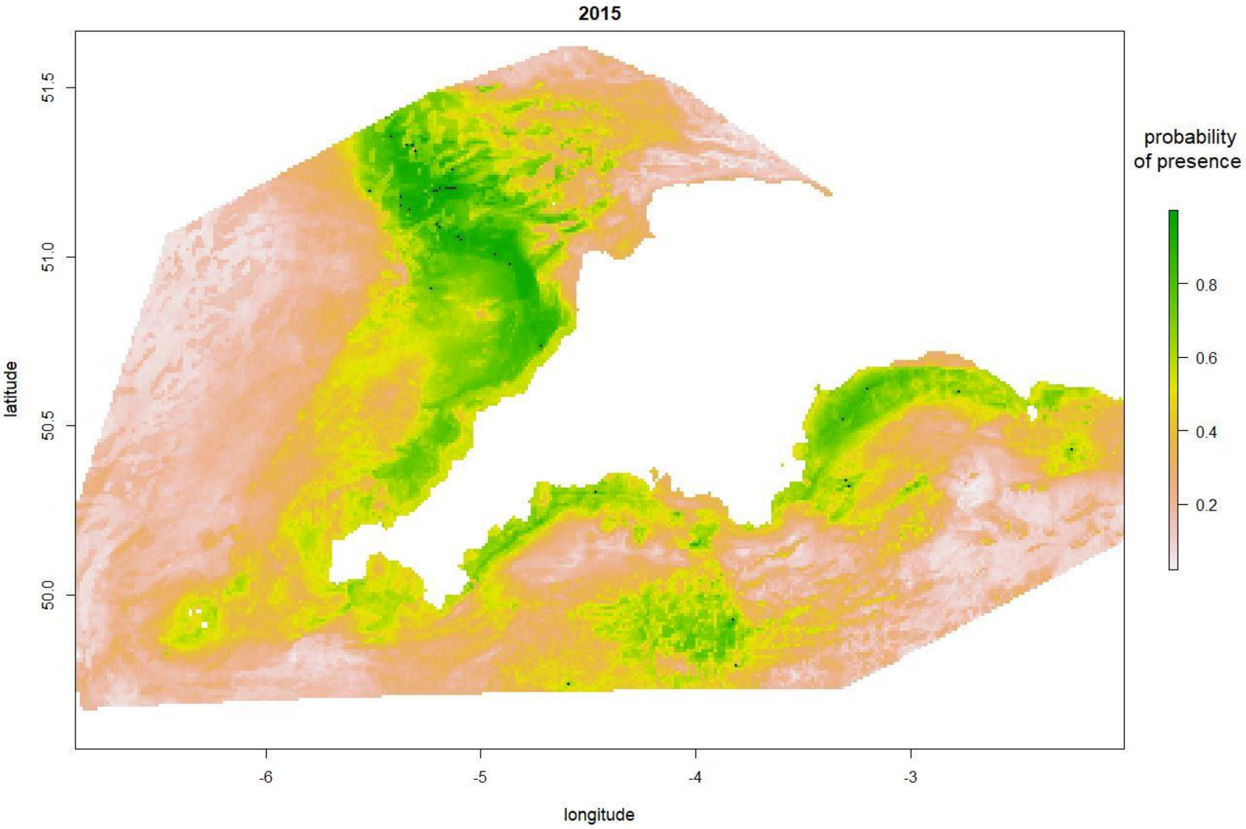
2015
latitude
longitude
51.5
51.0
50.5
50.0
-6
-5
-4
-3
probability
of presence
0.8
0.6
0.4
0.2

(d)

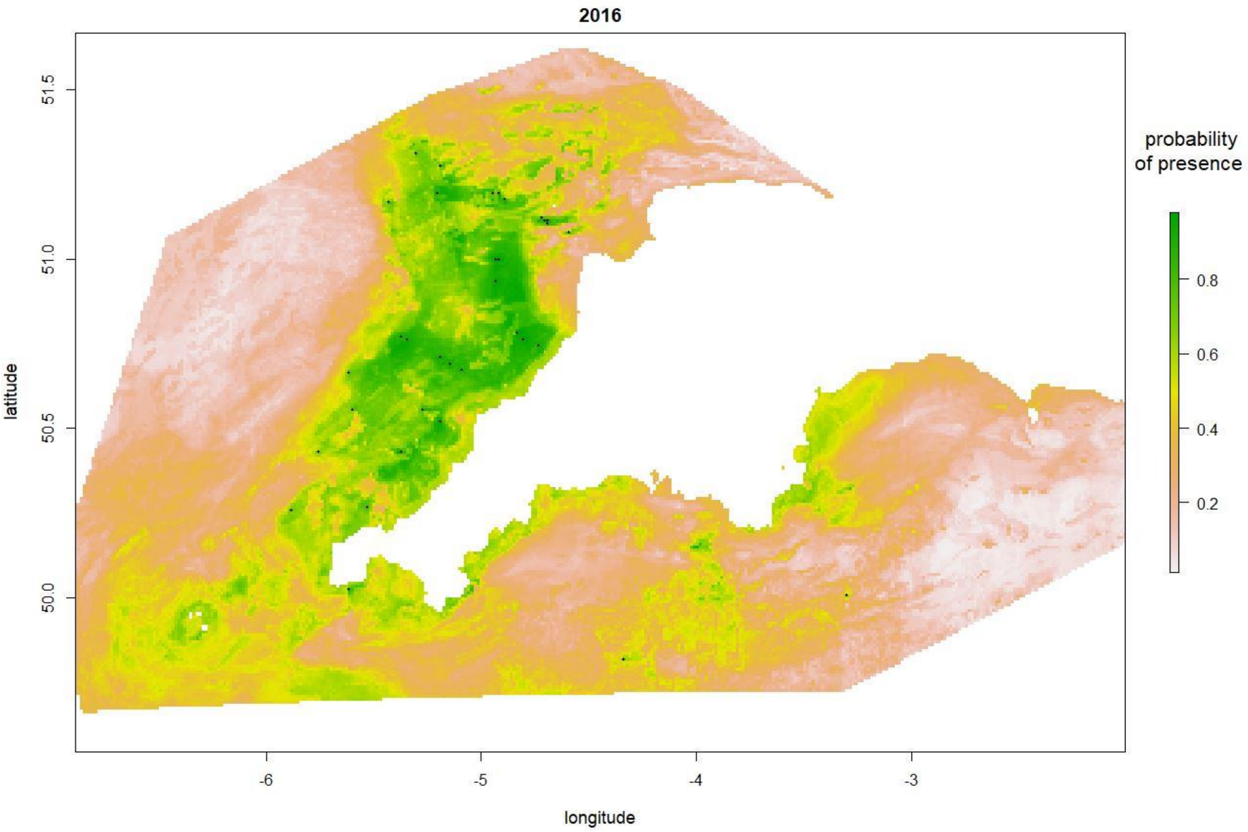
2016
probability
of presence
0.8
0.6
0.4
0.2
51.5
51.0
50.5
50.0
latitude
-6
-5
-4
-3
longitude

(e)

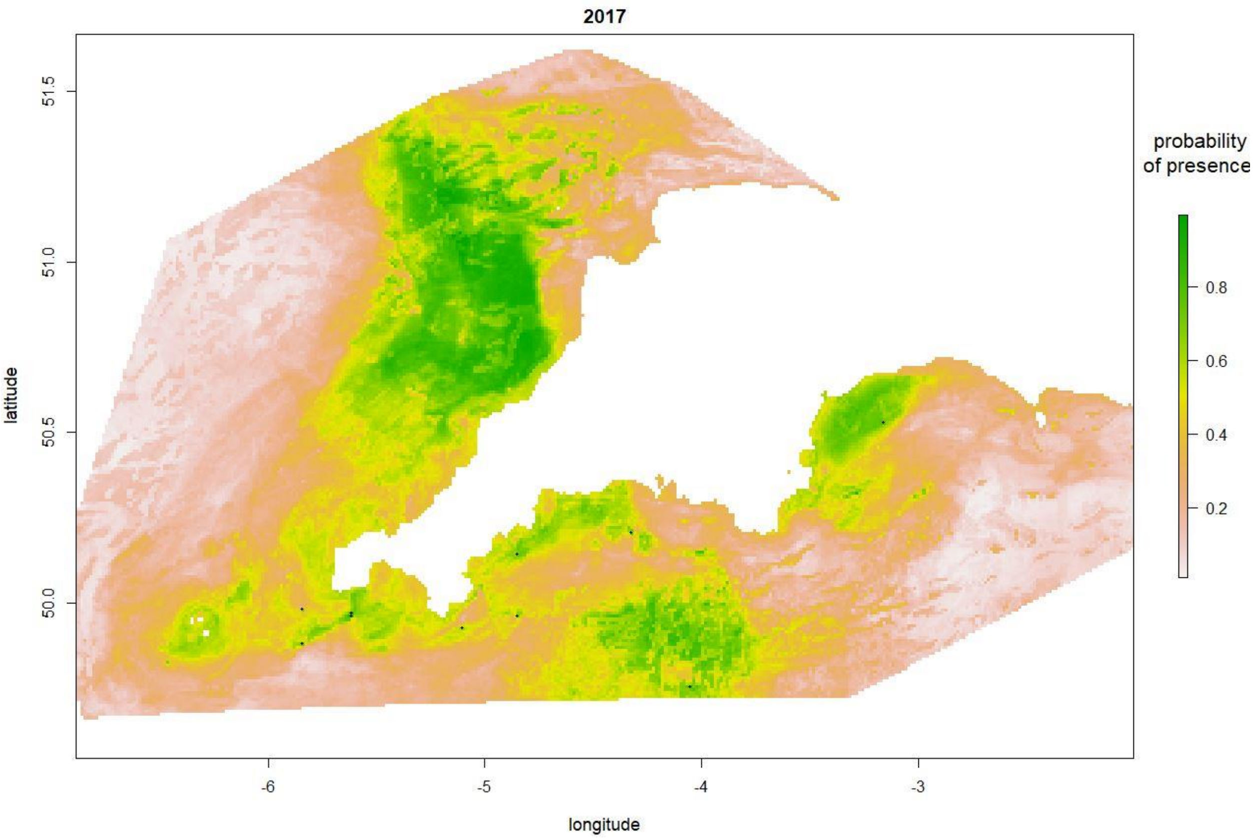
2017
latitude
51.5
51.0
50.5
50.0
longitude
-6
-5
-4
-3
probability
of presence
0.8
0.6
0.4
0.2

Figure S.3 Residuals of predictive GAM

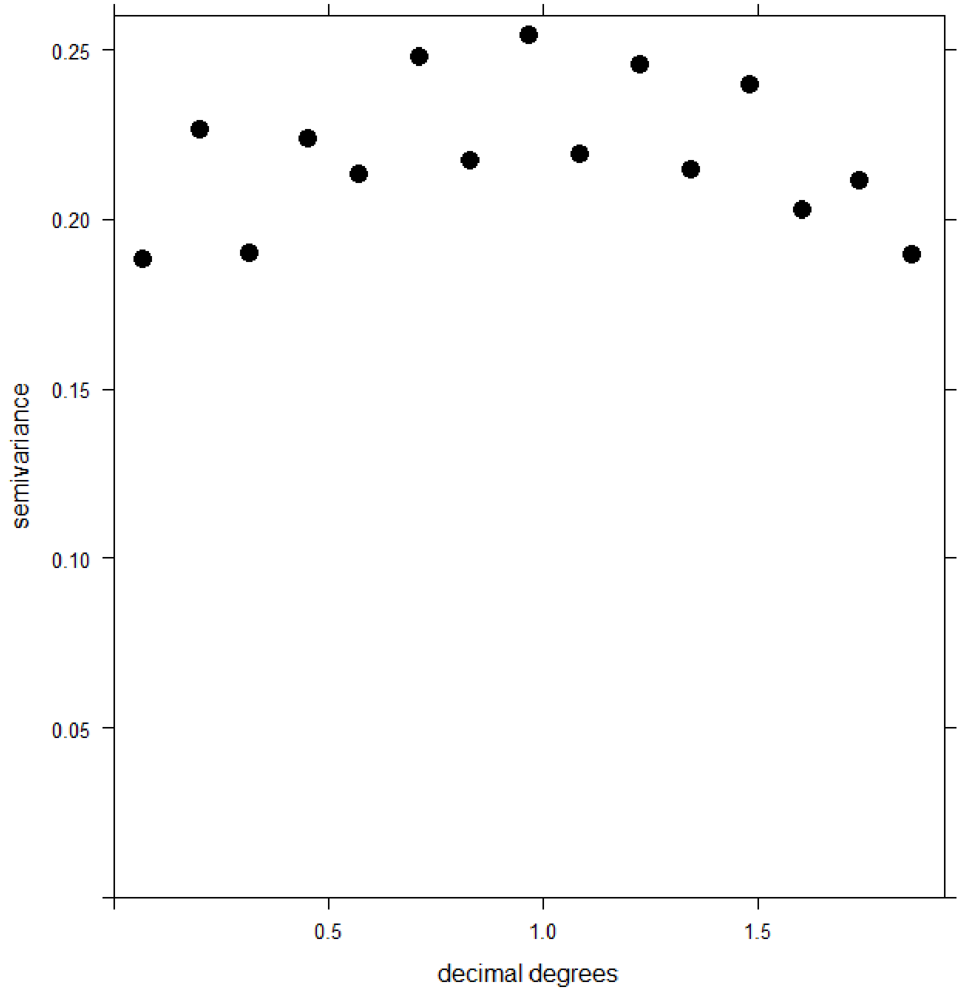

Appendix 2

Accuracy of data

We are confident that identification of Balearic shearwaters was accurate for several reasons. Firstly, most (96%) Balearic shearwaters sightings were in flight, when identification is much easier due to their underwing plumage and body shape (Gil-Velasco, Rodriguez, Menzie, & Arcos, 2015). Secondly, the classic decline in detectability with distance was seen for all Balearic shearwaters recorded in these surveys: 48% of sightings were within 300 m of the vessel, 71% within 500 m, and 93% within 800 m. Thirdly, all observers were trained, experienced marine surveyors who rated their identifications as 'definite' or 'probable'. In 2017, when other shearwater species (Manx, sooty and great) were all recorded, 94% of Balearic shearwater identifications were classified as 'definite'.

Continuous recording of birds in flight

Relatively low densities of Balearic shearwaters were detected during surveys over the five years, and only 393 could be included in our analysis. To improve detection of at-sea aggregations, it was necessary to include all observations recorded out to 1 km whilst the vessel was not stationary (defined as spending over 15 minutes in a 1km^2 cell) or steaming above 17 knots. To prevent inflation of bird abundance estimates, conventional ESAS methods recommend restricting the analysis of birds in flight to those recorded in 300 m square 'snapshots'. However, in this case the very low density of Balearic shearwaters made the danger of inflation of Balearic shearwater abundance estimates small (especially because we used bespoke methods to continuously record observed birds in flight) compared to the benefit of including birds observed out to 1,000 m in snapshot counts. Additionally, because we only used these data for observations up to 1000m from the vessel in models to predict probability of Balearic shearwater *presence* (not to estimate Balearic shearwater abundance) and to identify key environmental predictors of Balearic shearwater occurrence, we believe it appropriate to use all available sightings of the species in the models.

Figure S.4 boat speed of all Balearic shearwater sightings included in the analysis

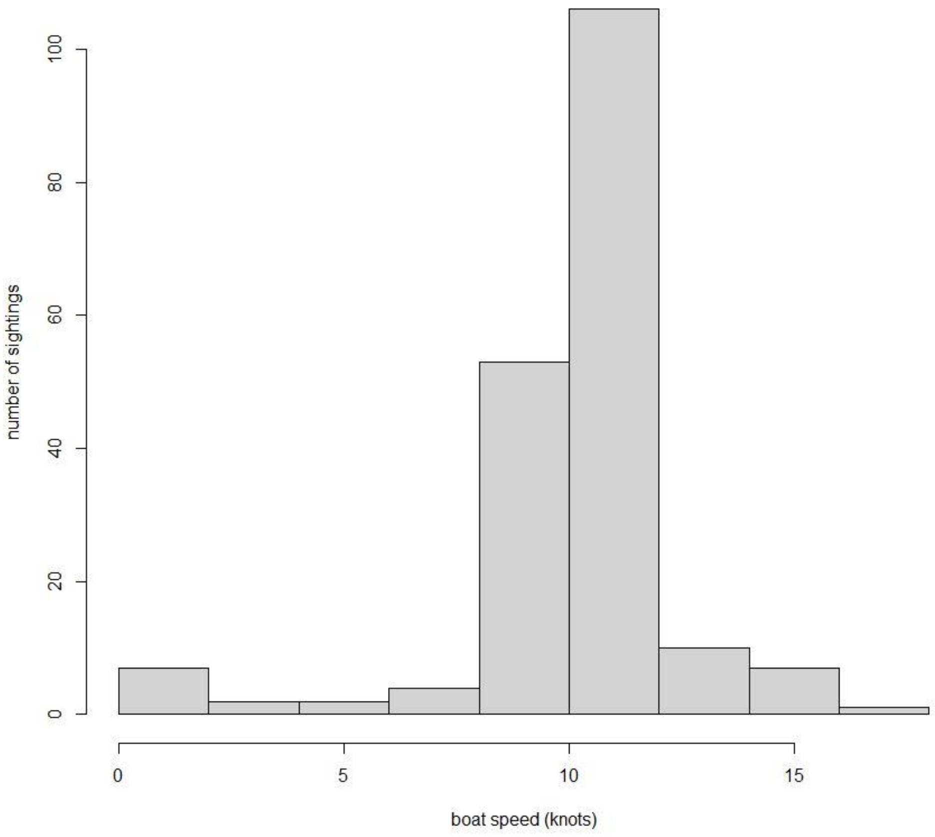

Figure S.5 triangulated distance to boat transects of all raw sightings

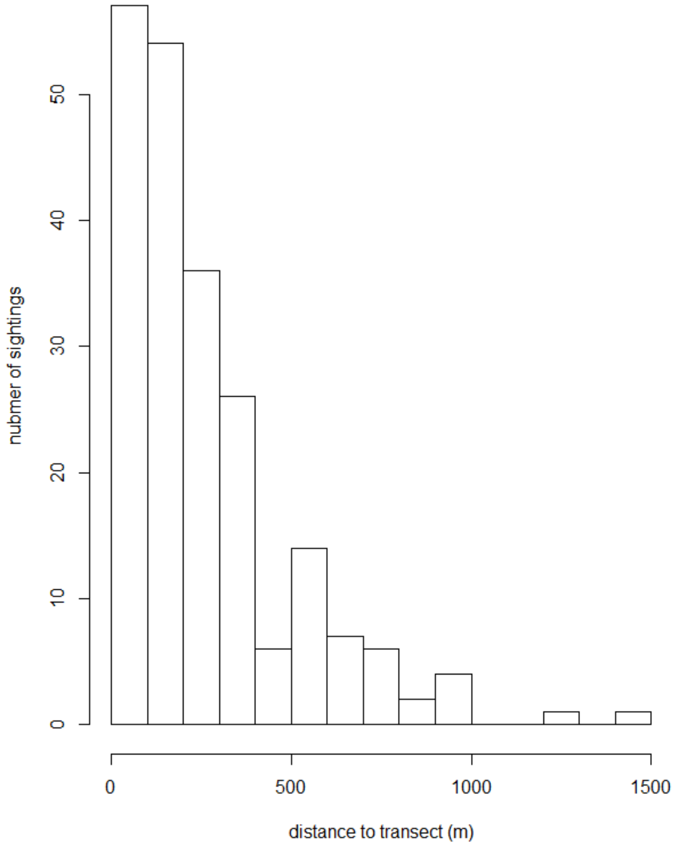

Supplement: Supplementary file 1 — Appendix [file ECE3-11-1544-s001.pdf]
